# ZL006, a small molecule inhibitor of PSD-95/nNOS interaction, does not induce antidepressant-like effects in two genetically predisposed rat models of depression and control animals

**DOI:** 10.1371/journal.pone.0182698

**Published:** 2017-08-03

**Authors:** Sandra Tillmann, Vitor Silva Pereira, Nico Liebenberg, Anne Karina Christensen, Gregers Wegener

**Affiliations:** Department of Clinical Medicine, Translational Neuropsychiatry Unit, Aarhus University, Aarhus, Denmark; Klinikum der Johann Wolfgang Goethe-Universitat Frankfurt, GERMANY

## Abstract

N-methyl-D-aspartate receptor (NMDA-R) antagonists and nitric oxide inhibitors have shown promising efficacy in depression but commonly induce adverse events. To circumvent these, a more indirect disruption of the nitric oxide synthase/postsynaptic density protein 95 kDa complex at the NMDA-R has been proposed. This disruption can be achieved using small molecule inhibitors such as ZL006, which has attracted attention as ischemic stroke therapy in rodents and has been proposed as a potential novel treatment for depression. Based on this, our aim was to translate these findings to animal models of depression to elucidate antidepressant-like properties in more detail. In the present study, we administered ZL006 to two established animal models of depression and control rodents. Following treatment, we measured locomotion in the Open Field and depressive-like behavior in the Forced Swim Test and Tail Suspension Test. Our experimental designs included the use of different species (rats, mice), strains (Flinders Sensitive Line rats, Flinders Resistant Line rats, Wistar Kyoto rats, Wistar Hanover rats, Sprague Dawley rats, B6NTac mice), routes of administration (intraperitoneal, intracerebroventricular), times of administration (single injection, repeated injections), treatment regimens (acute, sustained), and doses (5, 10, 15, 50 mg/kg). ZL006 did not affect behavior in any of the described settings. On a molecular level, ZL006 significantly reduced total nitrate/nitrite concentrations in the cerebellum, supporting that it is capable of reducing nitric oxide metabolites in the brain. Future studies using different experimental parameters are needed to further investigate the behavioral profile of ZL006.

## Introduction

Depression is a devastating psychiatric disorder with a lifetime prevalence of 16% and estimated annual costs of €92 billion in Europe [1–3], and is expected to become the leading cause of the global disease burden by 2030 [4]. Despite the availability of several classes of antidepressant drugs, current monoamine treatment options are inadequate due to a delayed onset of action, a high nonresponse rate (> 30%), and severe adverse effects [5, 6]. These findings stress the urgent need to develop novel therapeutic targets outside the classic monoamine pathway with high efficacy and low side effect profiles.

Growing evidence highlights the relevance of the glutamatergic system, since administration of ketamine, a non-selective N-methyl-D-aspartate receptor (NMDA-R) antagonist, led to rapid, long-lasting improvement in depressed patients [7–10]. However, ketamine exerts severe side effects, such as psychosis, psychotomimetic effects, and chronic neurotoxic effects, limiting its use in a clinical environment [9, 11–13]. A more selective agent would ideally retain ketamine’s antidepressant effects without exhibiting its adverse effects [10]. Nitric oxide (NO) is a small, highly diffusible gas molecule synthesized from L-arginine by nitric oxide synthase (NOS) [14, 15]. Activation of the NMDA-R and subsequent Ca^2+^ influx triggers Ca^2+^-calmodulin to bind to NOS, which in turn produces NO. The most extensively characterized downstream signaling pathway of NO is soluble guanylate cyclase, which catalyzes the synthesis of cGMP from GTP [15]. Several studies suggested that the L-arginine-NO-cGMP pathway may mediate the actions of antidepressant agents. For example, the antidepressant effects of escitalopram [16], duloxetine [17], topiramate [18], and lamotrigine [19] all seem to depend on inhibition of either NMDA-R or NO-cGMP synthesis. Moreover, several antidepressants were shown to inhibit hippocampal NOS activity, which may largely involve NMDA-R-related mechanisms [20]. A recent study suggested that NO is involved in the antidepressant effect of ketamine, as L-arginine pre-treatment prevented the antidepressant action of ketamine. Furthermore, ketamine was found to reduce cNOS activity in the hippocampus [21]. Clinical data revealed elevated plasma NO metabolite concentrations in suicidal patients as well as increased NO production in depressed patients [22]. Correspondingly, decreasing or blocking NOS activity (and hence NO synthesis) induced antidepressant-like effects in several animal studies (reviewed in [15, 23]). Despite these promising leads, even selective nNOS inhibitors exhibit adverse effects [11, 24, 25], excluding sole NOS inhibition as an antidepressant target. Therefore, more targeted downstream mediators of NMDA-Rs and nNOS must be taken into consideration.

In order to interact with the NMDA-R, nNOS is anchored to the membrane by the scaffolding protein PSD-95, enabling downstream signaling via the carboxy-terminal PDZ ligand of nNOS [26]. Disruption of this complex specifically prevents NMDA-R signaling coupled to nNOS, while leaving other functions of both the NMDA-R and nNOS intact. To achieve this, peptide fragments and small molecule inhibitors have been employed, such as 4-(3,5-Dichloro-2-hydroxy-benzylamino)-2-hydroxybenzoic acid (ZL006). ZL006 attenuated neurological deficits in ischemic stroke models without inhibiting NMDA-R function, catalytic activity of nNOS, or spatial memory [27]. While NMDA-R antagonists may impair source memory in rats, ZL006 did not affect memory or motor function, confirming a more favorable therapeutic outcome of PSD-95/nNOS disruption [28]. Moreover, it was suggested that ZL006 possesses antidepressant-like properties in CD-1 mice [11].

However, no study has translated behavioral or molecular effects of PSD-95/nNOS disruption to an animal model of depression, although such models are indispensable tools for an advanced understanding of the underlying pathophysiology of the disease and subsequent treatment strategies. The aim of the present study was therefore to investigate behavioral effects of ZL006 in Flinders Sensitive Line (FSL) and Wistar Kyoto rats, both validated genetic animal models of depression [29–34], and their respective control strains, Flinders Resistant Line (FRL) and Wistar Hanover rats. In addition, we explored antidepressant-like effects in Sprague Dawley rats and B6NTac mice.

## Materials and methods

### Animals

Male Sprague Dawley rats (experiment 1) were purchased from Taconic Bioscience A/S (Ry, Denmark). Male Wistar Kyoto/Wistar Hanover rats (experiment 6; originally derived from Charles River, Sulzfeld, Germany) and male Flinders Sensitive Line (FSL)/Flinders Resistant Line (FRL) rats (experiments 2–3, 5, 7–8; originally derived from the colony at the University of North Carolina, USA) were taken from the colony maintained at Aarhus University, Denmark. All rats (aged 8–13 weeks) weighed 203–457 g (*M* = 321 g, *SD* = 53.1) at the start of experimental procedures and were pair-housed in standard cages (Cage 1291H Eurostandard Type III H, 425 × 266 × 185 mm, Tecniplast, Italy) at 20 ± 2°C and 60 ± 5% relative humidity on a 12 h light/dark cycle (lights on at 06:00 a.m.). Rats used in experiment 7 were housed in individual cages after surgery. Male B6NTac mice with a C57BL/6 background (experiment 4, aged 10–12 weeks) were obtained from Taconic Bioscience A/S (Ry, Denmark) and weighed 21.5–31.6 g (*M* = 26.24 g, *SD* = 2.44) at the start of experimental procedures. Mice were group-housed (n = 6–7 per cage) in standard cages and kept in scantainers on the same light cycle as rats. Tap water and chow pellets were available to all animals *ad libitum*; along with access to a tunnel shelter, nesting material, and a wooden stick. Animals were allowed to acclimatize for 1 week and were weighed once a week. Rats were additionally handled every day for at least 3 days prior to behavioral experiments. All experiments were approved by the Danish Animal Experiments Inspectorate (2012-15-2934-00254) prior to initiation of the experiments and were conducted in accordance with the European Communities Council Directive.

### Drugs

The small molecule inhibitor ZL006 was obtained from WuXi AppTec (Shanghai, China) and injected systemically (intraperitoneal (i.p.), experiments 1–6, 8) or centrally (intracerebroventricular (i.c.v.), experiment 7) at different doses. For systemic injections at doses between 5 and 50 mg/kg (based on [11]), ZL006 was dissolved in 0.9% saline at pH 12 before adjusting it back to physiological levels (pH 7.4). Control animals were injected with saline, which was pH-adjusted in the same manner. For central infusions at doses of 10–100 μg/5 μL, ZL006 was dissolved in 35% Dimethyl sulfoxide (DMSO, D5879-1L, Sigma Aldrich, St. Louis, MO, USA) diluted in saline. Consequently, vehicle for control animals consisted of a mixture of 35% DMSO in saline. Imipramine hydrochloride (15 mg/kg, i.p.) was obtained from Sigma Aldrich (Taufkirchen, Germany) and dissolved in saline.

The following drugs were used for the surgical procedures described in experiment 7: fentanyl-fluanisone (Hypnorm, VetaPharma Ltd, Leeds, UK; 0.1 mg/kg fentanyl citrate and 3.3 mg/kg fluanisone; i.p.), midazolam (Hameln Pharmaceuticals GmbH, Hameln, Germany; 5 mg/kg; i.p.), lidocaine (FarmaPlus, Oslo, Norway; 10 mg/kg; s.c.), carprofen (Rimadyl, Pfizer Inc., NY, USA; acquired from Orion Pharma, Nivå, Denmark; 5 mg/kg; s.c.), buprenorphine (Temgesic, RB Pharmaceuticals Ltd, Berkshire, UK; 0.1 mg/kg; s.c.), ampicillin (PharmaCoDane ApS, Herlev, Denmark; 333 mg/kg; s.c.). All drugs were freshly prepared on the day of the experiment.

### Surgical procedure

Stereotactic surgical procedures were performed under semi-sterile conditions as described previously [35]. Prior to surgery, rats were anesthetized with fentanyl-fluanisone and midazolam until reflexes were absent. After animals were unresponsive, they were mounted onto a stereotactic frame (Kopf Instruments, Tojunga, CA, USA) and given carprofen. To numb the skin, a total volume of 0.15 mL/rat of lidocaine was injected locally. After making the incision, a flat skull orientation (i.e., bregma and lambda lie in the same coronal plane) was achieved by setting the incision bar at −3.3 mm. Rats were placed on a heating pad throughout surgery to ensure a constant body temperature. An i.c.v. guide cannula (21 G, 12 mm length) was inserted 2 mm above the right lateral ventricle (coordinates from bregma: AP: −1.0 mm, ML: −1.6 mm, DV: −3.0 mm [36]), anchored to the skull with two stainless steel screws and dental acrylic (GC Corporation, Tokyo, Japan), and closed by a dummy stylet. Immediately following surgery, rats received injections of buprenorphine, ampicillin, and saline (10 mL/kg; s.c.). Animals, previously pair-housed, were housed singly after surgery, allowed to recover for 7 days prior to drug infusions, and handled daily for 5 days prior to the testing day to habituate them to the injection procedure. They were monitored closely (twice daily) in the period between surgeries and injections to ensure their wellbeing. No additional analgesics were given following the carprofen administration after surgery, as no animal showed any signs of distress or pain in the postsurgical period.

### Infusion and verification of cannula placement

On the testing day, rats were randomized to be infused with vehicle (5 μL of a mixture of 35% DMSO in saline) or ZL006 (experiment 7a: 10 μg/5 μL; experiment 7b: 100 μg/5 μL) dissolved in 35% DMSO diluted in saline. Injections were performed in conscious rats loosely restrained by the experimenter as previously experienced during handling. Drugs were slowly infused through an infusion cannula which extended 1 mm beyond the guide cannula. The injection rate was 2 μL/min and the cannula remained in place for an additional 30 seconds to allow diffusion. Behavioral testing took place 19–30 min following infusions (time intervals in min; infusion: 00:00–00:03, OF: 00:22–00:27, FST: 00:28–00:33). The correct placement of the cannula was verified via dye injection after decapitation through visual inspection. Animals with incorrect cannula placement (n = 2) were excluded from further analysis.

### Behavioral experiments

All behavioral procedures were performed in specially-equipped rooms within the animal facility between 08:00 a.m. and 12:00 p.m. Animals were moved into behavioral rooms at least 1 h before testing to allow habituation.

#### Open field

Locomotion was measured in an open field (OF) arena (100 × 100 × 80 cm, 10 lx) as previously described [37]. Animals were placed in the center of the square and allowed to move freely for 5 min. The total distance travelled was recorded by a camera mounted to the ceiling and scored with specialized software tracking the midpoint of the rats’ body contour (Noldus Ethovision XT, Wageningen, Netherlands).

#### Forced Swim Test

To measure depressive-like behavior, the modified Forced Swim Test (FST) [38] was employed. Rats were placed into a perspex cylinder (height 60 cm, diameter 24 cm) filled with 24 (± 1°C heated tap water to a height of 40 cm. After 5 min of swimming, rats were removed from the water, dried with towels and returned to their home cages. Water was changed after each session. Rat behavior was recorded by a camera positioned in front of the four tanks. In some experimental conditions, a pretest of 15 min was conducted 24 h before the test session to accentuate behavioral responses following drug treatment [38, 39]. Three distinct parameters were scored by an observer blinded to treatment with a time-sampling technique, whereby the predominant behavior over each 5-s period of the 300-s test was rated [39]. Behaviors distinguished were struggling, indicated by vertical climbing movements of the forepaws, usually against the wall of the swim cylinder and typically breaking the surface of the water; swimming, with horizontal movements throughout the cylinder (including diving); and immobility, characterized by a floating upright posture, where the rat only makes small movements to keep its head above the water surface. Struggling is usually categorized as escape-directed behavior and interpreted as active stress coping behavior, whereas immobility is seen as passive stress coping behavior and considered to reflect depressive-like symptoms [38].

#### Tail Suspension Test

To assess depressive-like behavior, mice underwent the Tail Suspension Test (TST) as originally described [40]. Briefly, mice were suspended by their tails from a horizontal rod using adhesive tape attached approximately 1 cm from the tip of the tail. To establish baseline immobility, a pre-TST was performed before drug injections, followed by testing sessions of the same duration (6 min). Two distinct parameters (struggling, immobility) were scored by an observer blinded to treatment with a time-sampling technique whereby the predominant behavior over each 5-s period of the 360-s test was rated. Mice were considered immobile when they hung passively and completely motionless, indicating passive stress coping behavior. Conversely, motion was scored as struggling, suggesting escape-directed and antidepressant-like behavior.

### Nitrate/Nitrite measurements

To measure nitrate/nitrite concentrations in the cerebellum, we performed an enzyme-linked immunosorbent assay (ELISA) according to the manufacturer’s instructions (Nitrate/Nitrite Fluorometric Assay Kit #780051, Cayman Chemical, MI, USA). Upon decapitation, the cerebellum was separated from the brain, immediately snap-frozen using pre-cooled isopentane, and stored at −80°C until further use. Cerebellar tissue was homogenized and prepared as a 4× w_mean_/v dilution in 1× PBS + 1:25 proteinase inhibitor cocktail (Roche Diagnostics GmbH, Mannheim, Germany).

### Data analysis

Differences between treatment groups were assessed using a variety of tests, depending on the experiment. In single-strain experiments, a one-way analysis of variance (ANOVA) test was performed. In experiments using more than one strain, a two-way ANOVA was carried out. Multiple within-subject tests were analyzed using repeated-measures ANOVA. If any statistically significant differences were found following ANOVA, post-hoc comparisons using Bonferroni were performed to determine the direction of significance. Nonsignificant interaction effects were omitted from the result section. Assumptions of normality and homogeneity of variances were tested using Shapiro-Wilk test and Levene’s test, respectively, and were met by all data sets. Data in the figures are depicted as means ± standard error of the mean (SEM); alpha was set at .05. All statistics were performed using IBM SPSS 22.0 (IBM Corp., Armonk, NY, USA) and GraphPad Prism 5.0 (GraphPad Software Inc., San Diego, CA, USA).

### Experimental design

#### Experiment 1. Dose-dependent systemic effects of ZL006 in Sprague Dawley rats

To establish the optimal dose of ZL006 in rats, we initially used Sprague Dawley rats, a normal (i.e., nondepressed) reference rat strain with similar dose responses to FSL rats. Sprague Dawley rats (n = 32) were randomized to receive ZL006 (5, 10, or 50 mg/kg) or vehicle (saline) and were exposed to a 15-min pre-FST 24 h prior to the testing day. On the testing day, rats were injected i.p. with either ZL006 or vehicle. The OF was conducted 50 min post-injection for a duration of 5 min, followed by the FST performed 60 min post-injection for 5 min. One week later, rats were again subjected to a 5-min FST session to investigate potential sustained effects. Dose selections were based on a study indicating that 10 mg/kg of ZL006 had antidepressant-like properties in mice [11].

#### Experiment 2a. Acute systemic effects of ZL006 in FSL/FRL rats

To test antidepressant effects of ZL006 in an animal model of depression, FSL (n = 14) and FRL (n = 14) rats were randomized to receive ZL006 (15 mg/kg) or vehicle (saline). The dose was based on Experiment 1, showing that 50 mg/kg elicited locomotor effects, which was not the case at 10 mg/kg. We therefore increased the dose minimally (to 15 mg/kg) to elicit a behavioral response without locomotor effects. Upon i.p. drug administration, rats were tested in the OF (5 min, 50 min post-injection) and FST (5 min, 60 min post-injection). No pre-FST was performed, as FSL rats show sufficient immobility without pre-exposure [31]. After 24 h, rats were again subjected to a 5-min FST session.

#### Experiment 2b. Replication of experiment 2a with additional higher dose

FSL (n = 24) and FRL (n = 22) rats were randomized to receive ZL006 (15 or 50 mg/kg) or vehicle (saline). After i.p. injections, they were tested in the OF (5 min, 50 min post-injection) and the FST (5 min, 60 min post-injection).

#### Experiment 3. Sustained systemic effects of ZL006 in FSL/FRL rats

To exclude any effects of habituation, pre-OF and pre-FST tests were performed in FSL (n = 14) and FRL (n = 14) rats randomized to ZL006 (15 mg/kg) or vehicle (saline). All rats were exposed to the OF for 15 min to habituate them to the arena. Immediately afterwards, they underwent a pre-FST session for 15 min to establish baseline immobility. One hour after the pre-FST, they were injected i.p. with ZL006 or vehicle. After 24 h and 72 h, they were again subjected to the OF and FST for 5 min each. This protocol was based on the aforementioned study reporting antidepressant effects of ZL006 in mice after habituation to behavioral tests [11].

#### Experiment 4. Sustained systemic effects of ZL006 in B6NTac mice

This experiment was conducted to explore the effects of ZL006 in mice (n = 26) receiving ZL006 (10 mg/kg) or vehicle (saline). Mice were exposed to a 15-min pre-OF to habituate them to the arena. Immediately afterwards, they underwent a 6-min pre-TST to establish baseline immobility. After the pre-TST, they were injected i.p. with ZL006 or vehicle. After 24 h and 72 h following injections, they were again subjected to the OF (5 min) and TST (6 min).

#### Experiment 5. Systemic effects of repeated injections of ZL006 in FSL/FRL rats

To investigate the effects of repeated injections of ZL006, FSL (n = 20) and FRL (n = 26) rats were randomized to receive i.p. injections of ZL006 (15 mg/kg), imipramine (serving as a positive control, 15 mg/kg), or vehicle (saline) at three different time points (24 h, 19 h, and 1 h prior to the FST [41]). All rats were subjected to a 15-min pre-FST session 24 h prior to the testing session. Injections were performed immediately after the pre-FST.

#### Experiment 6. Systemic effects of repeated injections of ZL006 in FSL/FRL and Wistar Kyoto/Wistar Hanover rats

To investigate the effects of repeated injections of ZL006 in a different animal model of depression, Wistar Kyoto (n = 16) and Wistar Hanover (n = 8) rats were randomized to receive i.p. injections of ZL006 (10 mg/kg) or vehicle (saline) at three different time points (24 h, 19 h, and 1 h prior to the FST). All rats were subjected to a 15-min pre-FST session 24 h prior to the testing session. Injections were performed immediately after the pre-FST. FSL (n = 16) and FRL (n = 8) rats were included as additional groups and underwent the same procedure.

#### Experiment 7a. Central effects of ZL006 in FSL rats

To explore the effects of central administration compared with systemic administration, FSL (n = 13) and FRL (n = 6) rats underwent a surgical procedure in which a guide cannula was stereotactically implanted into the right lateral ventricle. After a 7-day recovery period, ZL006 (10 μg/5 μL) or vehicle (5 μL saline), both dissolved in 35% DMSO, were perfused through the guide cannula at a rate of 2 μL/min and were held in place for 30 sec afterwards to allow diffusion. After 19 and 25 min following infusions, rats underwent a 5-min OF test and a 5-min FST, respectively.

#### Experiment 7b. Replication of experiment 7a with a 10 × higher dose

To investigate whether central effects of ZL006 may be dose-dependent, we replicated experiment 7a with FSL (n = 17) and FRL (n = 9) rats receiving a higher dose of ZL006 (100 μg/5 μL); all other parameters were equal to experiment 7a.

#### Experiment 8. Cerebellar nitrate/nitrite measurements after repeated ZL006 injections in FSL rats

To assess the capability of ZL006 to decrease NO metabolites, we analyzed total nitrate/nitrite and nitrite concentrations in the cerebellum using an ELISA assay. Behaviorally naïve FSL rats (n = 16) were randomized to receive i.p. injections of ZL006 (10 mg/kg) or vehicle (saline) at three different time points (24 h, 19 h, and 1 h prior to decapitation). After decapitation, the cerebellum was taken out, immediately snap-frozen in pre-cooled isopentane and stored at −80°C until further use.

## Results

### Experiment 1. Dose-dependent systemic effects of ZL006 in Sprague Dawley rats

**Fig 1:**
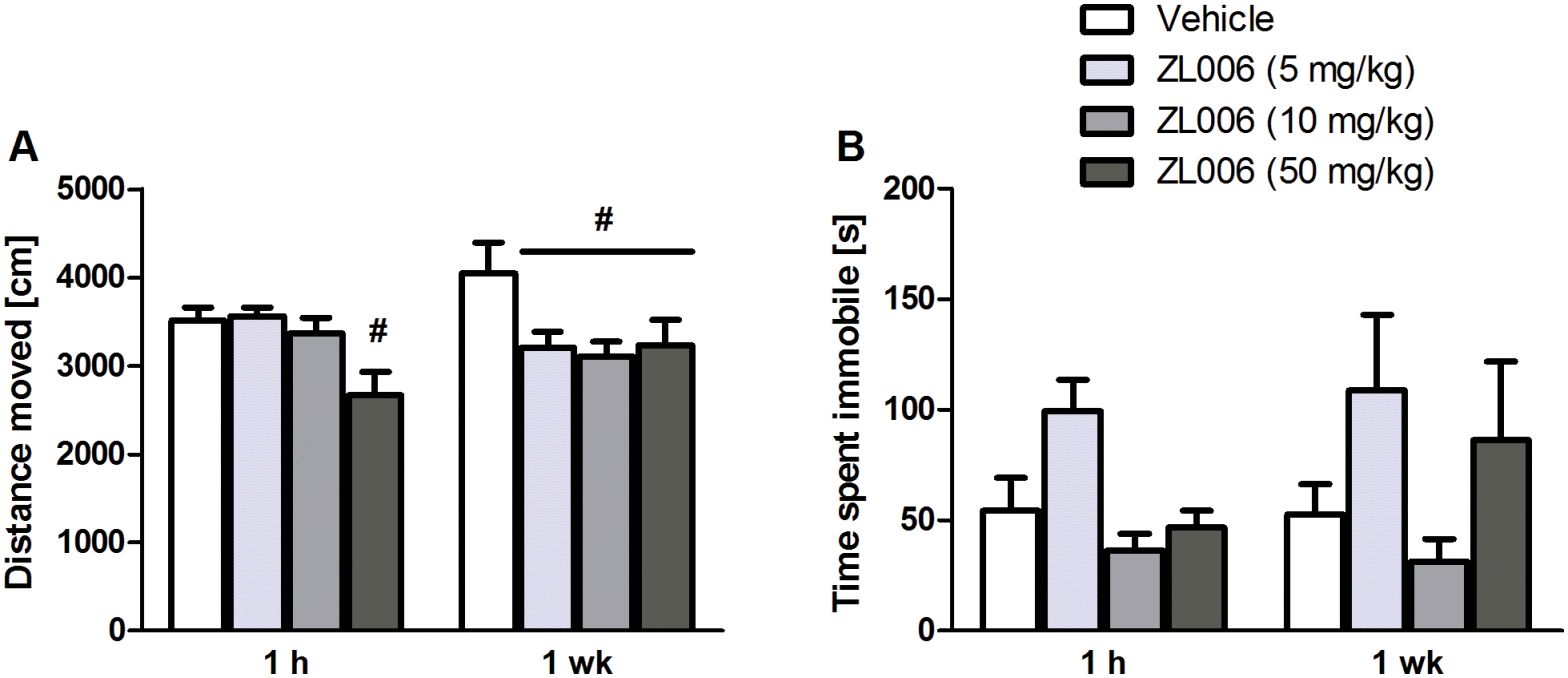
Dose-dependent systemic effects in Sprague Dawley rats 1 h and 1 week after injection of vehicle or ZL006 (5, 10, 50 mg/kg). **A**: Locomotion during a 5-min Open Field session. After 1 h, animals receiving 50 mg/kg ZL006 moved significantly less compared with those treated with vehicle. After 1 week, this effect was present in all ZL006-injected rats; #: *p ≤* .05 compared with respective vehicle; n = 6–8/group. **B**: Immobility during a 5-min Forced Swim Test session. There were no significant strain or treatment effects, *p*’s > .05; n = 6–8/group. Values are expressed as means ± SEM and were analyzed using one-way ANOVAs followed by Bonferroni post-hoc tests.

Treatment groups significantly differed in the distance travelled in the OF after 1 h (*F*(3,28) = 5.164, *p* = .006, one-way ANOVA). Post-hoc analysis revealed that rats receiving 50 mg/kg ZL006 moved significantly less compared with vehicle (*p* = .017; Fig 1A). After 1 week, this effect was present in all ZL006-treated rats (*p*’s < .05). There were no significant effects in the FST (*p*’s > .05, two-way mixed ANOVA; Fig 1B).

### Experiment 2a. Acute systemic effects of ZL006 in FSL/FRL rats

**Fig 2:**
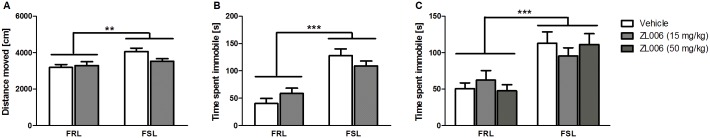
Acute systemic effects in Flinders Sensitive/Resistant Line rats 1 h after injection of vehicle or ZL006 (15 mg/kg (B); 15–50 mg/kg (C)). **A**: Locomotion during a 5-min Open Field session. A significant strain difference indicated that FSL rats moved a greater total distance than FRL rats; **: *p* ≤ .005; n = 7/group. **B**: Immobility during a 5-min Forced Swim Test session. A significant strain difference indicated that FSL rats were more immobile than FRL rats; ***: *p ≤* .001; n = 7/group. **C**: Replication of B with the inclusion of an additional dose (50 mg/kg). A significant strain difference indicated that FSL rats were more immobile than FRL rats; ***: *p ≤* .001; n = 6–8. Values represent means ± SEM and were analyzed using two-way ANOVAs followed by pairwise comparisons.

There was a significant strain effect in the OF 1 h after injection (*F*(1, 24) = 9.715, *p* = .005, two-way ANOVA; Fig 2A), in that FSL rats moved more than FRL rats. Treatment did not significantly affect locomotion. In the FST, FSL rats showed significantly greater immobility than FRL rats (*F*(1, 24) = 47.31, *p* < .001, two-way ANOVA; Fig 2B). Struggling and swimming data showed the same pattern with main effects of strain but no significant treatment or interaction effects (*p*’s > .05). Retesting the animals after 24 h yielded the same results as described after 1 h.

### Experiment 2b. Replication of experiment 2a with additional higher dose

In the OF, FSL rats moved a greater distance than FRL rats (*F*(1,40) = 26.824, *p* < .001, two-way ANOVA). In the FST, FSL rats were more immobile than FRL rats (*F*(1, 40) = 27.590, *p* < .001, two-way ANOVA; Fig 2C). There were no significant treatment effects in either test (*p*’s > .05).

### Experiment 3. Sustained systemic effects of ZL006 in FSL/FRL rats

**Fig 3:**
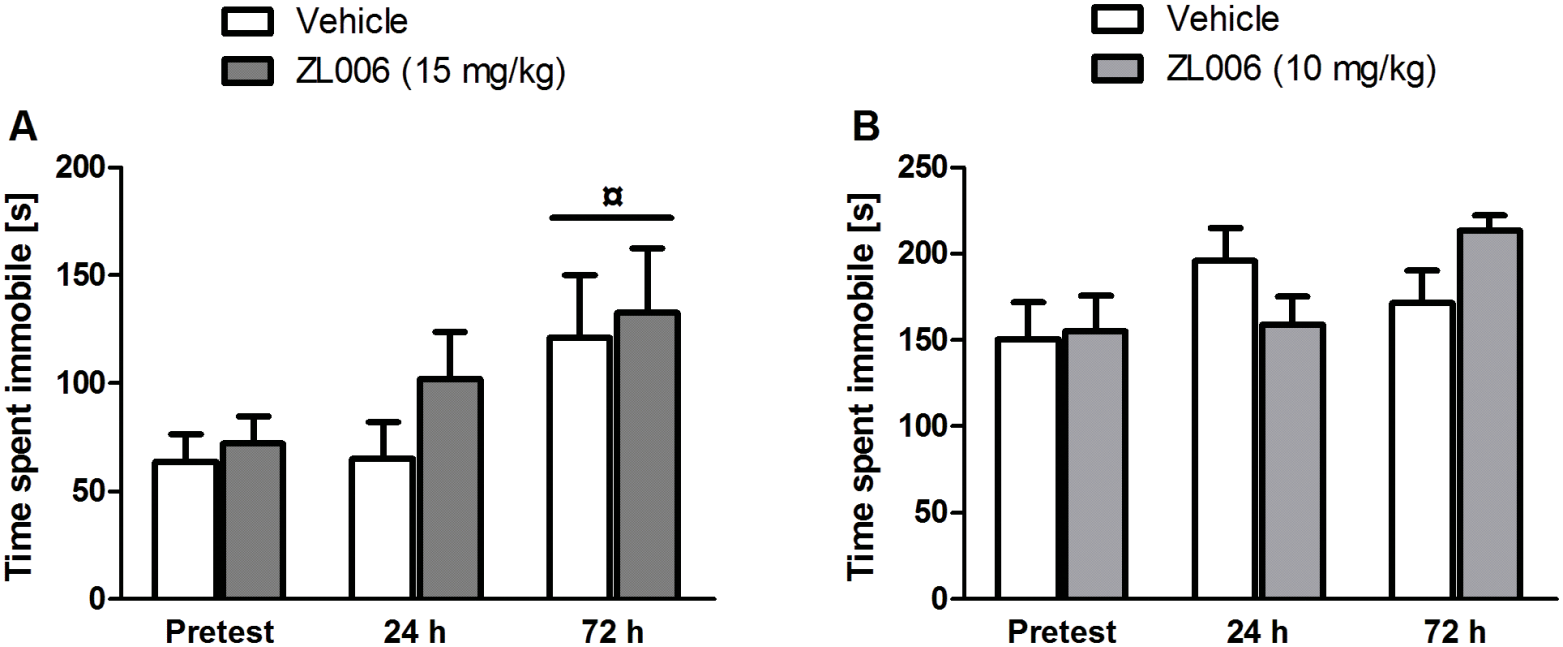
Sustained effects of ZL006 in Flinders Sensitive Line rats and B6NTac mice. **A**: Immobility of Flinders Sensitive Line rats in the Forced Swim Test at baseline (15 min pretest) and at 5-min test sessions 24 h and 72 h after injection of vehicle or ZL006 (15 mg/kg). Rats were more immobile 72 h after injections; ¤: *p ≤* .05 compared with the pretest; n = 7/group. **B**: Immobility of B6NTac mice in 6-min Tail Suspension Test sessions at baseline (pretest) and 24 h and 72 h after injection of vehicle or ZL006 (10 mg/kg); n = 11–13/group. Values are expressed as means ± SEM and were analyzed using a three-way mixed ANOVA (A) and one-way ANOVA (B) followed by pairwise comparisons.

There were no strain or treatment effects in the OF after 24 or 72 h (*p’s* > .05, two-way ANOVA). In the FST, there was a Time × Strain interaction (*F*(2,40) = 4.928, *p* = .012, three-way mixed ANOVA). Pairwise comparisons showed that FSLs were more immobile 72 h after injections compared with the pretest (*p* = .022; Fig 3A), which was not the case for FRL rats (*p* > .05). As expected, mean immobility scores in the FST were higher in FSL than in FRL rats (*F*(1,20) = 27.343, *p* < .001, three-way mixed ANOVA). No significant treatment effects were found (*p*’s > .05).

### Experiment 4. Sustained systemic effects of ZL006 in B6NTac mice

Locomotion in the OF did not differ between treatment groups (*p* > .05, one-way ANOVA). In the TST, two mice in the vehicle group were removed from further analysis after video inspection because they climbed their tails for more than 5% of the time period analyzed. There were no significant treatment effects (*p*’s > .05, two-way mixed ANOVA; Fig 3B).

### Experiment 5. Systemic effects of repeated injections of ZL006 in FSL/FRL rats

**Fig 4:**
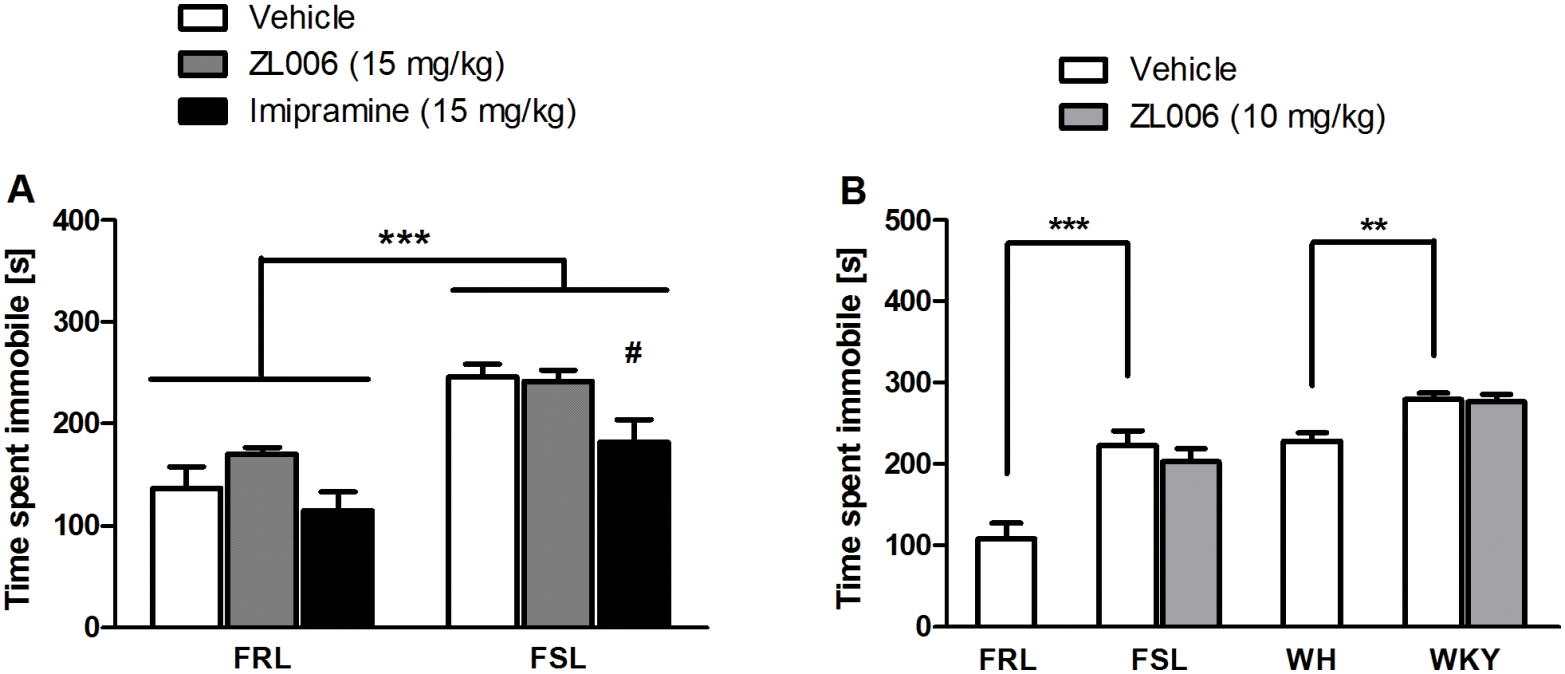
Systemic effects of repeated injections of ZL006 in Flinders Sensitive/Resistant Line and Wistar Kyoto/Wistar Hanover rats. **A**: Effects of ZL006 in FSL/FRL rats in a 5-min Forced Swim Test session after repeated injections (24 h, 19 h, 1 h prior to the FST) of vehicle, ZL006 (15 mg/kg), or imipramine (15 mg/kg). A significant strain difference indicated that FSL rats were more immobile than FRL rats; ***: *p ≤* .001. Moreover, imipramine-treated FSL rats were significantly less immobile than vehicle-treated FSL rats; #: *p ≤* .05; n = 6–10/group. **B**: Effects of ZL006 in FSL, FRL, Wistar Kyoto (WKY), and Wistar Hanover rats (WH) after repeated injections (24 h, 19 h, 1 h prior to the FST) of vehicle or ZL006 (10 mg/kg). When comparing vehicle-treated rats, FSL rats were more immobile than FRL rats, and Wistar Kyoto rats were more immobile than Wistar Hanover rats; **: *p ≤* .005; ***: *p ≤* .001; n = 8/group. Values are expressed as means ± SEM and were analyzed using a two-way ANOVA (A) and two separate one-way ANOVAs (B) followed by pairwise comparisons.

FSL rats moved a greater distance in the OF than FRL rats (*F*(2, 45) = 3.86, *p* = .028, two-way ANOVA). Moreover, FSL rats treated with imipramine showed decreased locomotion compared with vehicle (*F*(2, 45) = 4.37, *p* = .018, two-way ANOVA). In the FST, FSL rats were more immobile than FRL rats (*F*(1, 40) = 35.924, *p* < .001, two-way ANOVA; Fig 4A). Moreover, there was a main effect of treatment (*F*(2, 40) = 6.521, *p* = .004), in that imipramine significantly reduced immobility time (*p* = .048, Bonferroni correction). There were no significant effects of ZL006 in either test (*p*’s > .05).

### Experiment 6. Systemic effects of repeated injections of ZL006 in FSL/FRL and Wistar Kyoto/Wistar Hanover rats

Two separate one-way ANOVAs were performed in FSL/FRL rats and Wistar Kyoto/Wistar Hanover rats. In the OF, vehicle-treated FSL rats moved more than FRL rats (*F*(1, 14) = 11.346, *p* = .005), and vehicle-treated Wistar Kyoto rats moved less than Wistar Hanover rats (*F*(1, 14) = 32.258, *p* < .001). In the FST, vehicle-treated FSL rats were more immobile than FRL rats (*F*(1, 14) = 19.198, *p* = .001), and vehicle-treated Wistar Kyoto rats were more immobile than Wistar Hanover rats (*F*(1, 14) = 14.447, *p* = .002; Fig 4B). There were no significant treatment effects in either test (*p*’s > .05).

### Experiment 7a. Central effects of ZL006 in FSL rats

**Fig 5:**
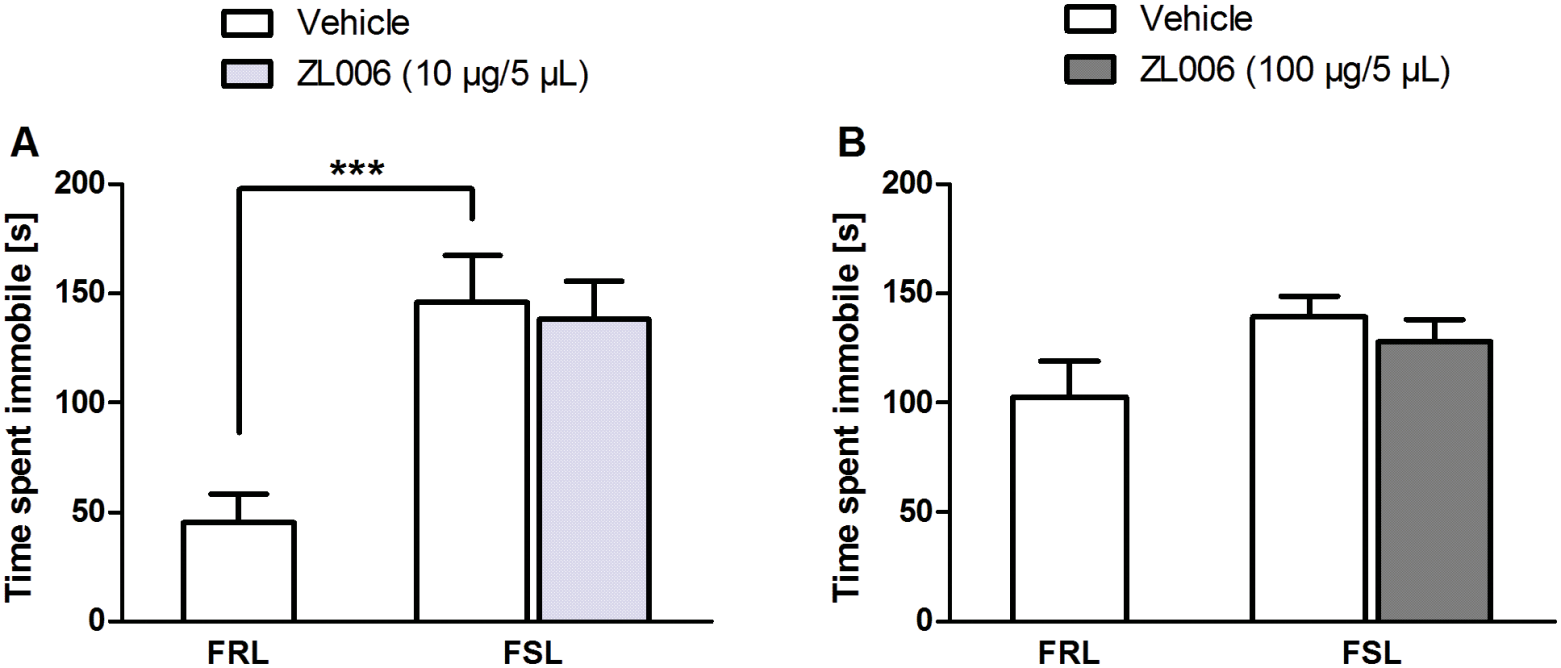
Central effects of ZL006 in Flinders Sensitive/Resistant Line rats. **A**: Immobility of Flinders Resistant Line (only received vehicle) and Flinders Sensitive Line rats during a 5-min FST session 25 min following infusion of vehicle or ZL006 (10 μg/5 μL) at a rate of 2 μL/min. A significant strain difference indicated that vehicle-treated FSL rats were more immobile than FRL rats; **: *p ≤* .005; n = 5–6/group. **B**: Replication of A with a 10 × higher dose (100 μg/5 μL). There were no significant strain or treatment effects, *p*’s *>* .05; n = 8–9/group. Values represent means ± SEM and were analyzed using one-way ANOVAs followed by pairwise comparisons.

Two animals were excluded from further analysis due to incorrect placement of the cannula and bending of the cannula in the cage. In the OF, vehicle-treated FSL rats moved more than FRL rats (*F*(1, 9) = 6.579, *p* = .030, one-way ANOVA). In the FST, vehicle-treated FSL rats were more immobile than FRL rats (*F*(1, 9) = 17.185, *p* = .003, one-way ANOVA; Fig 5A). There were no significant effects of treatment (*p*’s > .05).

### Experiment 7b. Replication of experiment 7a with a 10 × higher dose

One FRL rat was excluded from the analysis due to bending of the needle. Vehicle-treated FSL rats moved more in the OF than FRL rats (*F*(1, 14) = 12.518, *p* = .003, one-way ANOVA), but there was no significant treatment effect. There were no strain or treatment effects in the FST (*p*’s > .05; Fig 5B).

### Experiment 8. Cerebellar nitrate/nitrite measurements after repeated ZL006 injections in FSL rats

**Fig 6:**
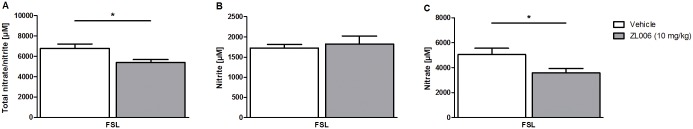
Cerebellar nitrate/nitrite measurements after repeated ZL006 (10 mg/kg) injections (24 h, 19 h, and 1 h prior to decapitation) in Flinders Sensitive Line rats. **A**: Total nitrate/nitrite concentrations were decreased in rats treated with ZL006 compared to vehicle, *: *p ≤* .05. **B**: Nitrite concentrations were unchanged between treatment groups, *p >* .05. **C**: Nitrate concentrations ([total nitrate/nitrite]–nitrite) were decreased in ZL006-treated rats, *: *p ≤* .05; n = 8/group. Values represent means ± SEM and were analyzed using one-way ANOVAs.

Rats treated with ZL006 showed decreased total nitrate/nitrite levels compared with vehicle, (*F*(1, 15) = 6.804, *p* = .021, one-way ANOVA; Fig 6A). Nitrite levels were unchanged between treatment groups (*p* > .05; Fig 6B). Calculating nitrate levels ([total nitrate/nitrite]–nitrite) showed decreased nitrate levels in ZL006-treated rats (*F*(1, 15) = 5.807, *p* = .03; Fig 6C).

## Discussion

The present study did not find evidence for behavioral effects of ZL006 in two animal models of depression or control rodents, using different species, strains, routes of administration, times of administration, treatment regimens, and doses. Modulating glutamatergic neurotransmission with an NMDA-R antagonist like ketamine was previously found to have antidepressant-like properties in both rodents and humans [8, 42] but is not devoid of adverse effects [43]. We therefore took a more targeted approach by uncoupling nNOS from the scaffolding protein PSD-95, which may circumvent adverse events likely resulting from upstream targeting. Our study was based on previous work in mice [11], but no studies have addressed behavioral consequences of ZL006 in an animal model of depression.

We observed no acute behavioral effects (1 h after administration) of ZL006, which is in line with previous work [11]. Although the authors showed sustained effects after 24 h and 72 h, they did not report acute effects of ZL006. When they tested acute antidepressant-like effects of a structurally related compound (IC87201) in the TST after 1 h, no behavioral effects were visible, suggesting that small molecule inhibitors may substantially differ from the rapid-acting antidepressant effects of ketamine. In contrast with the previous study, our data did not confirm the reported sustained effects after 24 and 72 h. However, it should be noted that the authors only tested the effects of ZL006 in the TST, but not in the FST. Our study focused on the FST (except for experiment 4), since the TST can only be performed in mice. This may partly explain heterogeneous findings, since several studies reported marked differences between the two tests. Chatterjee et al. [44] found that acute single-dose injections of ketamine in mice increased the immobility duration in the TST but not FST, indicating enhanced sensitivity of the TST. Studies by Cryan et al. [45] and others [46] confirmed that FST and TST differ in terms of their response to drugs, in that the TST appears more sensitive. When the previous study [11] administered the related compound IC87201 and tested its effects in the FST, they did not find any acute behavioral effects either, which may lend partial support for our findings.

This study provides the first data on behavioral consequences of ZL006 in the FST, and is the first to test the compound in rats, hindering a direct comparison with previous studies. Rats and mice were previously shown to differ in cytochrome metabolism enzymes, which are essential for metabolizing the NMDA-R antagonist ketamine and may thus also be relevant for downstream NMDA-R inhibitors [47, 48]. In experiment 4, we administered ZL006 to mice, but did not yield the same results as the previous study. This may be due to a different strain used (inbred B6NTac mice with a C57BL/6 background vs. outbred CD-1 mice), although both strains are “normal” mice not exhibiting enhanced baseline immobility. Nevertheless, key mechanisms related to depression such as hippocampal cell proliferation and brain-derived neurotrophic factor (BDNF) expression levels were shown to differ between the two strains [49], potentially indicating important interstrain differences.

Importantly, we confirmed that ZL006 did not show any locomotor effects in rats at doses up to 15 mg/kg, which is in line with the aforementioned study. To our knowledge, this is the first study comparing central (i.c.v.) and systemic (i.p.) administration of ZL006. The majority of our experiments were conducted using systemic injections, as ZL006 was previously shown to readily cross the blood-brain barrier after systemic injection [50]. However, a recent paper reported low permeability of ZL006 through the blood-brain barrier [51]. To exclude any route-dependent mechanisms due to drug metabolism, we infused ZL006 centrally using two different doses, but did not see any differences compared with systemic administration. For comparative purposes, we included the tricyclic antidepressant imipramine as a positive control. As expected, imipramine reduced immobility of FSL rats in the FST, supporting the validity of our study results.

ZL006 was previously shown to suppress NMDA-stimulated cyclic guanosine monophosphate formation, indicative of decreased NO production [28]. To verify that ZL006 is capable of decreasing NO metabolite concentrations in the brain, we measured total nitrate/nitrite concentrations in the cerebellum. Nitrate/nitrite tissue concentrations were demonstrated to correlate closely with NOS activity in various brain regions [52]. The cerebellum was chosen because it produces the highest concentrations of NO within the brain [53]. While it might seem interesting to measure nitrate levels in limbic areas of the brain, previous studies showed that administration of NOS inhibitors produced a similar inhibition of NOS across various brain regions [52, 54]. It is therefore unlikely that nitrate/nitrite measurements in the hippocampus or prefrontal cortex would substantially differ from our results. We found that ZL006 reduced total cerebellar nitrate/nitrite concentrations compared with vehicle, which was mainly due to decreased nitrate. This finding suggests that ZL006 was able to modify NO metabolites in the brain, which strengthens the relevance of our findings. However, this decrease or the overall decrease in the whole brain may not have been large enough to elicit a behavioral effect or may precede a clinical behavioral manifestation.

Some limitations of this study deserve mentioning. First, while we did explore sustained effects after 24 h, 72 h, and 1 week, the study design could be improved by including separate groups for each time point to avoid carryover effects from previous swim sessions. Second, chronic treatment with ZL006 may reveal different effects, although repeated injections in our study did not affect immobility. While more than three injections may be deemed necessary to obtain an antidepressant effect, the need of chronic treatment would considerably contradict the concept of downstream NMDA mediators as rapid-onset antidepressants. Third, our study was not designed to enable a direct comparison between parameters, e.g. with regard to the effects of pre-swimming on acute vs. sustained effects of the drug. However, collectively, these individual experiments support the same conclusion, i.e., a lack of behavioral findings. Nevertheless, the list of parameters is not exhaustive, and further studies may show different results using different experimental designs, e.g., different animal models, behavioral tests, doses etc., which is beyond the scope of the current work.

The exact molecular mechanisms of the small molecule inhibitors ZL006 and IC87201 remain unclear. Previous studies largely ascribed the pharmacological effects of ZL006 to inhibition of the PSD-95/nNOS interaction [11, 27, 28, 55–60], in that ZL006 specifically binds to the extended nNOS-PDZ domain at the β-finger, thus preventing interaction with PSD-95 [61]. However, a recent study casted doubt on that explanation, as the authors found that neither ZL006 nor IC87201 interacted with the PDZ domains of nNOS or PSD-95, or inhibited the nNOS-PSD-95 interaction through the β-finger of nNOS-PDZ [61]. This is in contrast with the original study proposed by Zhou et al. [50] and challenges the viewpoint of small molecule inhibitors downstream of the NMDA-R. The consequences of this finding need to be further addressed in future studies, but could partly explain our results.

Finally, we cannot exclude any antidepressant properties of ZL006 in other experimental conditions, and encourage other researchers to use our study as a starting point for further investigating the properties of ZL006 with regard to psychiatric disorders.

## Supporting information

S1 Dataset Raw dataset for Fig 1. (XLSX)

S2 Dataset Raw dataset for Fig 2. (XLSX)

S3 Dataset Raw dataset for Fig 3. (XLSX)

S4 Dataset Raw dataset for Fig 4. (XLSX)

S5 Dataset Raw dataset for Fig 5. (XLSX)

S6 Dataset Raw dataset for Fig 6. (XLSX)
